# Prognostic Potential of Heart Rate and Hypertension in Multiple Myeloma Patients

**DOI:** 10.3389/fcvm.2021.681484

**Published:** 2021-09-27

**Authors:** Jie Wang, Manyun Tang, Yunxiang Long, Jingzhuo Song, Limei Chen, Mengchang Wang, Yongxin Li, Chaofeng Sun, Yang Yan

**Affiliations:** ^1^Atrial Fibrillation Centre and Department of Cardiovascular Medicine, The First Affiliated Hospital of Xi'an Jiaotong University, Xi'an, China; ^2^Department of Hematology, The First Affiliated Hospital of Xi'an Jiaotong University, Xi'an, China; ^3^Department of Hepatobiliary Surgery, The First Affiliated Hospital of Xi'an Jiaotong University, Xi'an, China; ^4^Department of Medical Oncology, The First Affiliated Hospital of Xi'an Jiaotong University, Xi'an, China; ^5^Department of Cardiovascular Surgery, First Affiliated Hospital of Xi'an Jiaotong University, Xi'an, China

**Keywords:** multiple myeloma, risk stratification, heart rate, hypertension, cardiac rhythm abnormalities

## Abstract

**Background:** The prognosis of patients with multiple myeloma (MM) is variable and partly depends on their cardiovascular status. The presence of arrhythmias can lead to worse outcomes. Therefore, this study aimed to evaluate the potential of heart rate (HR) and hypertension in predicating the outcomes of MM patients.

**Methods:** This study retrospectively enrolled patients with MM between January 1, 2010, and December 31, 2018, at the First Affiliated Hospital of Xi'an Jiaotong University. The endpoint was all-cause mortality. The Pearson's chi-square test was used to assess the association between hypertension and outcomes. Univariate and multivariate Cox proportional hazards models were developed to evaluate the relationship between HR and all-cause mortality.

**Results:** A total of 386 patients were included. The mean HR was 83.8 ± 23.1 beats per minute (bpm). Patients with HR >100 bpm had a higher all-cause mortality (79.4%, 50/63) than those with 60 ≤ HR ≤ 100 bpm (39.9%, 110/276) and <60 bpm (19.1%, 9/47) (*p* < 0.001). Subgroup analysis based on the International Staging System and sex revealed similar relationships (*p* < 0.01). When stratified by age, patients with HR >100 bpm had higher all-cause mortality than those with a lower HR when age was <65 years or 65–75 years (*p* < 0.001) but not >75 years. The proportion of patients with hypertension was 54.7% (211/386). However, hypertension was not associated with all-cause mortality in MM patients (χ^2^=1.729, *p* > 0.05). MM patients with HR >100 bpm had the highest all-cause mortality.

**Conclusions:** The prognostic potential of HR may be useful in aiding risk stratification and promoting the management of these patients.

## Introduction

Multiple myeloma (MM) is a malignant neoplasm characterized by the clonal proliferation of malignant plasma cells in the bone marrow and monoclonal protein in the blood or urine and is associated with organ dysfunction (1). The median age at diagnosis is 70 years, and approximately 62% of patients with MM are ≥65 years at the time of diagnosis (1, 2). Fortunately, the survival of patients with MM has improved dramatically over the past few decades because of therapeutic advancements (3, 4). Consequently, patients with increased age often have a high baseline incidence of coexisting cardiovascular diseases, including arrhythmias, hypertension, heart failure, and myocardial ischemia. In addition, MM is often associated with other chronic diseases, such as chronic kidney disease, which in turn leads to a high risk of cardiac events (5). Furthermore, chemotherapeutic agents for MM can give rise to treatment-related cardiotoxicities (6).

Heart rate (HR) is closely related to the development of cardiovascular morbidity and mortality in various diseases (7–9). Our recent work showed that some ECG parameters were related to the outcomes of MM (10), however, the association between HR, hypertension, and their prognostic potential in MM is rarely reported. Therefore, this study aims to assess the potential of HR and hypertension in predicating the outcomes of patients with MM.

## Methods

### Ethnic and Consent

This study was performed according to the Declaration of Helsinki with informed consent obtained from the patients or their family members for research purposes. The study protocol was approved by the Ethics Committee of the First Affiliated Hospital of Xi'an Jiaotong University (Approval No. XJTU1AF2020LSK-179).

### Participants and Groups

Consecutive MM patients were enrolled between January 2010 and December 2018 at the First Affiliated Hospital of Xi'an Jiaotong University. Multiple myeloma was diagnosed based on the results of testing and bone marrow pathology and confirmed by a hematologist. The treatment of MM patients was performed according to the new National Comprehensive Cancer Network (NCCN) guidelines (11). Based on HR, all patients were divided into three groups (HR >100 beats per minute (bpm), 60 ≤ HR ≤ 100 bpm, and HR <60 bpm). In this study, increased HR was generally defined as an HR over 100 bpm. All patients were divided into two groups according to baseline peripheral blood pressure (BP): hypertension (defined as having a systolic BP ≥140 mm Hg and/or a diastolic BP ≥90 mm Hg at baseline) and non-hypertension (12). Patients with missing information, a history of myocardial infarction, paced rhythm, or active infection (25 patients) and those who were lost to follow-up were excluded.

### Clinical Data Information

The baseline characteristics and clinical information and HR were collected from electronic medical records. HR was measured with a 12-lead electrocardiogram within 2 months of diagnosis for one patient and was obtained after at least 5 min of rest in the supine position during hospitalization; BP was measured at rest at least twice. Patients were stratified by the International Staging System (ISS) (13). All-cause mortality follow-up data were obtained from the data of survey participation until October 2019. The follow-up was conducted by outpatient visits, telephone calls, or other electronic media.

### Statistical Analysis

Continuous data were presented as mean ± standard deviation (SD) or median (interquartile range), and categorical variables were expressed as counts and percentages. The Pearson's chi-square test or Fisher's exact test was utilized for mortality to evaluate correlations between different groups. Survival curves were plotted with Kaplan–Meier analysis, and differences in survival were tested by the log-rank test. Univariate and multivariate Cox proportional hazards models were developed to identify the relationship between HR and all-cause mortality. The proportional hazards assumption was assessed. The software package SPSS version 24 (SPSS Inc., Chicago, IL, USA) was used for statistical analysis. A *p* < 0.05 was considered statistically significant.

## Results

### Baseline Characteristics

A total of 386 patients were enrolled in this study. The mean age of the patients was 61.7 ± 9.7 years, and 58.8% (227/386) were male. The median follow-up duration of 18.8 (9.5–36.8) months (Table 1), 169 patients (43.8%) died. The proportions of patients with HR >100 bpm, 60 ≤ HR ≤ 100 bpm and HR <60 bpm were 16.3% (63/386), 71.5% (276/386), and 12.2% (47/386), respectively. Sixty-three patients, including 17 patients with paroxysmal atrial fibrillation and four patients with paroxysmal supraventricular tachycardia, had increased HR. The proportion of patients with hypertension was 54.7% (211/386). One patient (0.3%, 1/386) was diagnosed with pulmonary embolism according to a CT pulmonary angiogram and received five cycles of a BCD regimen consisting of bortezomib, cyclophosphamide, and dexamethasone. The patient achieved complete remission and was alive through the end of follow-up. Nine patients (2.3%, 9/386) had heart failure, and 20 patients (5.2%, 20/386) had paroxysmal atrial fibrillation. Heart rate measurement data were obtained during sinus rhythm though they had the history of paroxysmal atrial fibrillation and supraventricular tachycardia in this study.

**Table 1:** Baseline characteristics of all patients.

**Patient demographics**	***N*** **= 386**
Age (years)	61.7 ± 9.7
Male, *n* (%)	227 (58.8)
Follow-up duration (months)	18.8 (9.5–36.8)
Systolic BP (mm Hg)	140.3 ± 19.1
Diastolic BP (mm Hg)	77.5 ± 12.6
Mean arterial pressure (mm Hg)	98.5 ± 13.3
Hypertension, *n* (%)	211 (54.7)
Log NT-proBNP (pg/mL)	3.06 ± 0.79
Troponin T (ng/mL)	0.280 (0.100–0.386)
Log lactate dehydrogenase (U/L)	2.38 ± 0.22
Serum albumin (g/L)	31.65 ± 6.05
Blood urea nitrogen (mmol/L)	6.92 (5.00–11.75)
Creatinine (mg/dL)	1.00 (0.69–2.57)
eGFR (mL/min/1.73 m^2^)	73.41 (24.74–99.32)
Calcium (mmol/L)	2.33 ± 0.39
Serum potassium (mmol/L)	3.55 ± 0.50
Serum sodium (mmol/L)	134.6 ± 5.8
**Complete blood count**	
White blood cell (×10^9^/L)	4.7 ± 2.5
Hemoglobin (g/L)	84.5 ± 23.5
Platelet (×10^9^/L)	157.0 ± 85.9
**Immune type of myeloma**, ***n*** **(%)**	
IgG/IgA/IgD	276 (71.5)
Light chain κ/λ	99 (25.6)
None secreted	11 (2.9)
**ISS Stage**, ***n*** **(%)**	
I	55 (14.3)
II	161 (41.7)
III	170 (44.0)
**HR (bpm)**	83.8 ± 23.1
<60, *n* (%)	47 (12.2)
60–100, *n* (%)	276 (71.5)
>100, *n* (%)	63 (16.3)
**Treatment**, ***n*** **(%)**	
ASCT	38 (9.8)
No ASCT	348 (90.2)
Bortezomib	142 (36.8)
Lenalidomide	34 (8.8)
Both bortezomib and lenalidomide	19 (4.9)
Conventional chemotherapy	186 (48.2)
Best supportive care	28 (7.3)

*Median (interquartile range), mean ± SD; eGFR, estimated glomerular filtration rate; ISS, International Staging System; ASCT, autologous stem cell transplantation; HR, heart rate; bpm, beats per minute; BP, blood pressure*.

### Prognostic Values of Heart Rate

**Figure 1:**
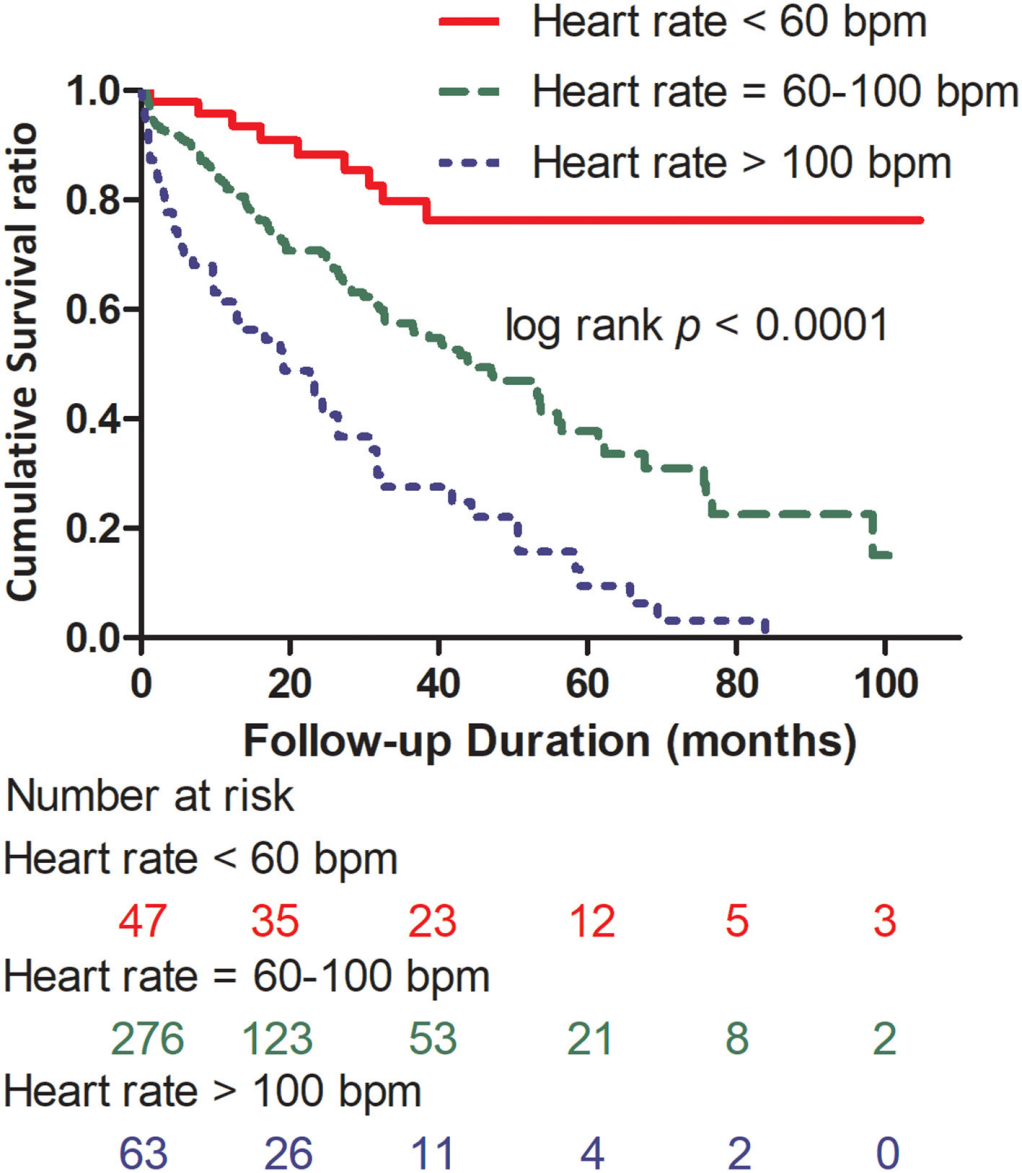
Kaplan–Meier survival curves according to the presence of heart rate >100 bpm, 60 ≤ heart rate ≤ 100 bpm, and heart rate <60 bpm, log-rank *p* < 0.0001. HR, heart rate; bpm, beats per minute.

The association between HR and all-cause mortality was evaluated. The mean HR of all patients was 83.8 ± 23.1 bpm. After several methods including the log-log survival curves, time-dependent Cox regression model and Schoenfeld residuals were used to test proportional hazards assumption for survival analysis, Cox model was identified to be suitable for the evaluation in our work, and the hazards were proportional. HR was independently associated with all-cause mortality (*p* < 0.01) (Table 2). Patients with HR > 100 bpm had a higher all-cause mortality (79.4%, 50/63) than those with 60 ≤ HR ≤ 100 bpm (39.9%, 110/276) and <60 bpm (19.1%, 9/47) (*p* < 0.001). HR showed a close association with all-cause mortality in the three groups (Figure 1).

**Table 2:** Univariate and multivariate analyses for overall mortality.

	**Univariate analysis**			**Multivariate analysis**		
	**Hazard ratio**	**95%CI**	***p*** **-value**	**Hazard ratio**	**95%CI**	***p*** **-value**
Age (years)	1.000	0.984–1.016	0.999			
Male sex	1.273	0.928–1.745	0.134			
Log NT-proBNP (pg/mL)	1.812	1.487–2.207	<0.001	1.163	0.911–1.485	0.224
Troponin T (ng/mL)	17.701	4.209–74.437	<0.001	3.112	0.527–18.361	0.210
Log lactate dehydrogenase (U/L)	3.220	1.827–5.673	<0.001	1.868	1.012–3.450	0.046
Serum albumin <35 g/L	1.195	0.847–1.686	0.309			
Blood urea nitrogen (mmol/L)	1.028	1.009–1.047	0.003	0.972	0.933–1.013	0.181
Creatinine (mg/dL)	1.105	1.057–1.156	<0.001	1.062	0.964–1.169	0.223
eGFR (mL/min/1.73 m^2^)	0.992	0.988–0.996	<0.001	0.999	0.992–1.006	0.759
Calcium (mmol/L)	1.087	0.751–1.574	0.658			
Serum potassium (mmol/L)	1.053	0.761–1.456	0.757			
Serum sodium (mmol/L)	0.944	0.918–0.971	<0.001	0.999	0.967–1.031	0.929
**Complete blood count**						
White blood cell (×10^9^/L)	1.098	1.041–1.158	0.001	1.075	1.018–1.135	0.009
Hemoglobin (g/L)	0.981	0.975–0.987	<0.001	0.988	0.980–0.997	0.007
Platelet (×10^9^/L)	0.999	0.997–1.001	0.477			
**ISS Stage**						
I	1			1		
II	1.956	1.053–3.632	0.034	1.324	0.685–2.561	0.404
III	3.385	1.854–6.179	<0.001	1.441	0.685–3.029	0.335
**HR (bpm)**						
<60	1			1		
60-100	3.436	1.737–6.799	<0.001	2.548	1.271–5.108	0.008
>100	8.087	3.961–16.510	<0.001	4.330	2.042–9.181	<0.001

*eGFR, estimated glomerular filtration rate; ISS, International Staging System; HR, heart rate; bpm, beats per minute*.

### Prognostic Value of Heart Rate Stratified by Sex

**Figure 2:**
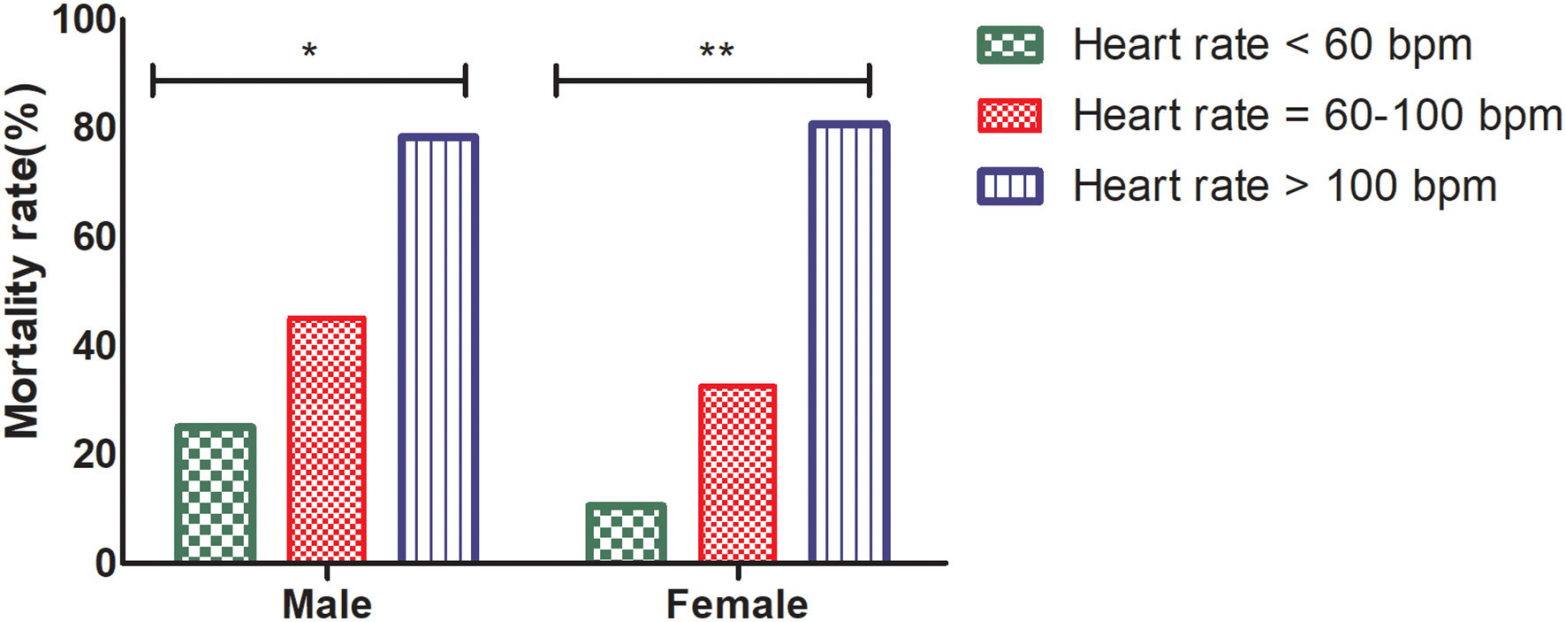
All-cause mortality of HR > 100 bpm, 60 ≤ HR ≤ 100 bpm, and HR <60 bpm in the male and female groups, Pearson's chi-square test, *p < 0.001, **p < 0.00001. HR, heart rate; bpm, beats per minute.

The mean age of male patients was 61.4 ± 10.0 years. Of the 227 male patients, the proportions of patients with HR >100 bpm was 16.3% (37/227), 60 ≤ HR ≤ 100 bpm was 71.4% (162/227), and HR <60 bpm was 12.3% (28/227). Patients with HR >100 bpm had higher all-cause mortality (78.4%, 29/37) than patients with 60 ≤ HR ≤ 100 bpm (45.1%, 73/162) and HR <60 bpm (25.0%, 7/28) (*p* < 0.001) in the male group. The mean age of female patients was 62.1 ± 9.4 years. Of the 159 female patients, the proportions of patients with HR > 100 bpm, 60 ≤ HR ≤ 100 bpm and HR <60 bpm were 16.4% (26/159), 71.7% (114/159), and 11.9% (19/159), respectively. Patients with HR > 100 bpm had higher all-cause mortality (80.8%, 21/26) than patients with 60 ≤ HR ≤ 100 bpm (32.5%, 37/114) and HR <60 bpm (10.5%, 2/19) (*p* < 0.00001) in the female group. In conclusion, HR showed prognostic potential in both the male and female subgroups (Figure 2).

### Prognostic Value of Heart Rate Stratified by ISS Stage

**Figure 3:**
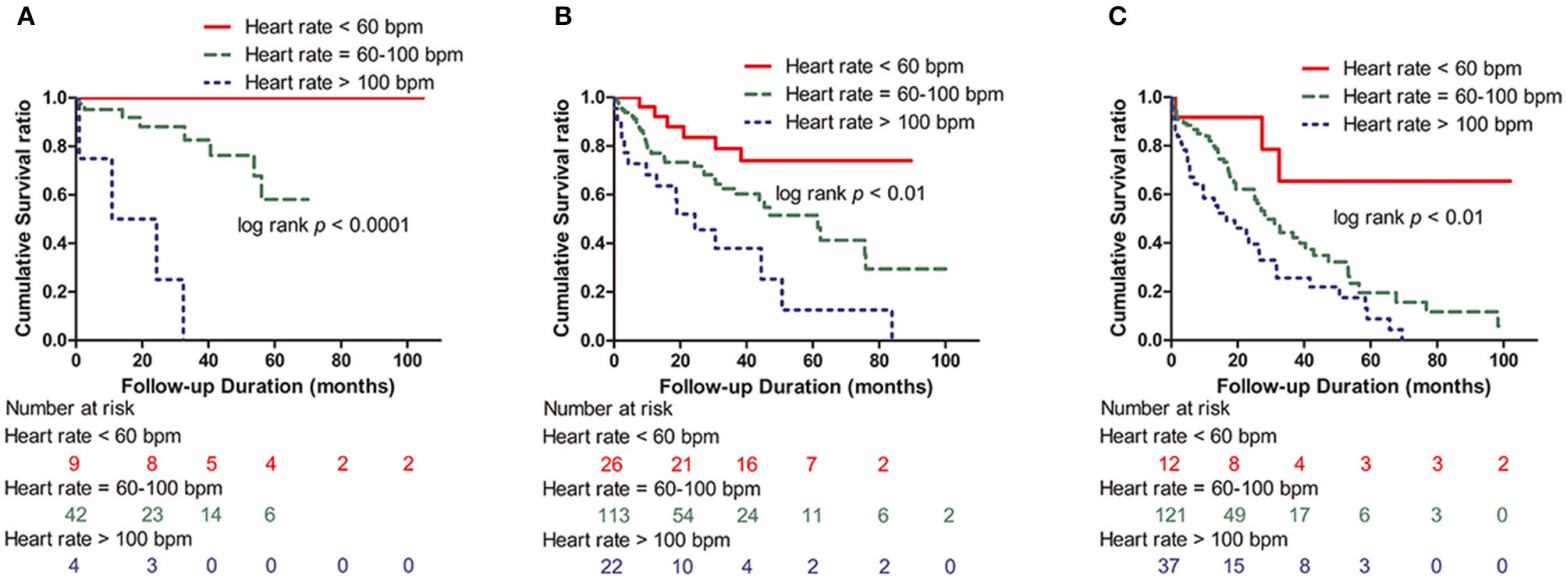
Kaplan–Meier survival curves according to the presence of HR >100 bpm, 60 ≤ HR ≤ 100 bpm, and HR <60 bpm in the ISS groups. **(A)** Stage I group (log-rank *p* < 0.0001). **(B)** Stage II group (log-rank *p* < 0.01). **(C)** Stage III group (log-rank *p* < 0.01); ISS, International Staging System; HR, heart rate; bpm: beats per minute.

The mean ages of patients were 59.5 ± 11.2, 61.7 ± 9.6, and 62.4 ± 9.3 years in Stages I, II, and III, respectively. Patients with Stage I, Stage II, and Stage III accounted for 14.3% (55/386), 41.7% (161/386), and 44.0% (170/386) of all patients. All-cause mortality of patients in Stage I, II, and III was 21.8% (12/55), 38.5% (62/161), and 55.9% (95/170), respectively. All-cause mortality of patients with HR >100 bpm, 60 ≤ HR ≤ 100 bpm and HR <60 bpm was 100.0% (4/4), 19.0% (8/42), and 0% (0/9) in Stage I; 68.2% (15/22), 36.3% (41/113), and 23.1% (6/26) in Stage II; and 83.8% (31/37), 50.4% (61/121), and 25.0% (3/12) in Stage III, respectively. In conclusion, patients with HR >100 bpm had higher all-cause mortality than patients with 60 ≤ HR ≤ 100 bpm and HR <60 bpm in all three ISS stages (*p* < 0.01) (Figure 3).

### Prognostic Value of Heart Rate Stratified by Age

**Figure 4:**
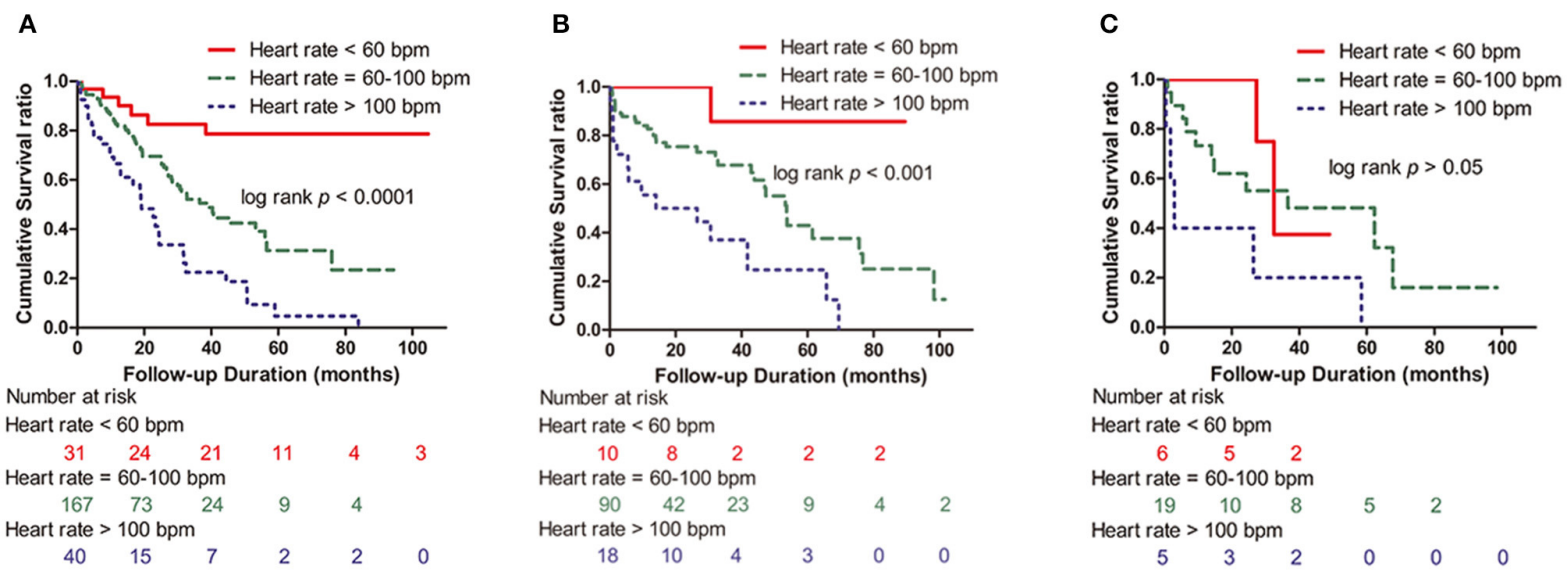
Kaplan–Meier survival curves according to the presence of HR >100 bpm, 60 ≤ HR ≤ 100 bpm, and HR <60 bpm in the age groups. **(A)** Age <65 years group (log-rank *p* < 0.0001). **(B)** Age 65–75 years group (log-rank *p* < 0.001). **(C)** Age >75 years group (log-rank *p* > 0.05). HR, heart rate; bpm, beats per minute.

In the age subgroup analysis, patients were divided into the following age groups: <65, 65–75, and >75. Patients <65 years old accounted for 61.7% (238/386), those 65–75 years old accounted for 30.5% (118/386), and those >75 years old accounted for 7.8% (30/386) of all patients. All-cause mortality in the age groups <65 years, age 65–75 years and age >75 years was 42.9% (102/238), 41.5% (49/118), and 60.0% (18/30), respectively. In the <65 year and 65–75 year groups, there was a significant correlation between all-cause mortality and HR (*p* < 0.0001, *p* < 0.001) (Figures 4A,B). In those aged >75 years, however, there was no statistical significance in all-cause mortality among the three groups of patients with HR > 100 bpm, 60 ≤ HR ≤ 100 bpm, and HR <60 bpm (log-rank *p* > 0.05) (Figure 4C).

### Prognostic Value of Hypertension

The mean arterial pressure was 98.5 ± 13.3 mm Hg. The proportion of patients with hypertension was 54.7% (211/386) in our study. The relationship between hypertension and all-cause mortality was evaluated. All-cause mortality in the hypertension and nonhypertension group was 40.8% (86/211) and 48.6% (85/175). However, hypertension was not associated with all-cause mortality in patients with MM (χ^2^=1.729, *p* > 0.05).

## Discussion

The prognostic potential of HR was evaluated in 386 patients with MM in our study. The findings indicated that HR was independently related to all-cause mortality in MM patients.

The prognosis of MM commonly depends on the identification of disease biology markers, patient factors, and cytogenetic classification (14–16). Accurate risk stratification and advancements in the management of MM have significantly increased survival and quality of life; however, many more cardiovascular complications are emerging (17). Drugs to treat MM show various cardiotoxicity profiles in MM patients. In addition, MM itself and related chronic diseases predispose patients to a high risk of cardiac events (18).

Cardiac arrhythmias are encountered frequently in patients with MM in clinical practice (19). Early identification of cardiac arrhythmias and burden is important for improving the prognosis of patients with MM. Previous studies have shown that fast HR is prospectively related to the development of cardiovascular morbidity and mortality and is an independent risk factor for atherosclerosis (7–9). Some important advantages of HR measurement are that it is less costly, noninvasive, and quick. This prompted us to perform this study with the main findings that fast HR was independently related to all-cause mortality in MM patients.

Arrhythmias are also the most common cardiac complications in patients with MM receiving chemotherapy, and the incidence of arrhythmias is considerably higher than the expected incidence for a given age. In a previous work, a total of 35,486 patients with MM were identified, of whom 31.2% had cardiac arrhythmias (19). MM patients have a heavy arrhythmia burden with a high risk of atrial fibrillation, which contributes to considerable hospitalization costs. Tachycardia was identified as a significant predictor of overall mortality (20). A total of 622 patients with cancer, including lung cancer, leukemia, lymphoma, or MM, were assessed for mortality adjusting for age and other factors that were significantly different between patients with an HR ≥100 bpm and those with an HR <100 bpm. In addition, cancer patients with experience tachycardia within 1 year after cancer diagnosis may have higher mortality rates up to 10 years after the diagnosis of tachycardia. Bradyarrhythmias and sinus node dysfunction have also been described in patients with MM undergoing chemotherapy (21, 22). A retrospective study indicated that MM patients using thalidomide had an HR of <60 bpm during follow-up, and 19% of thalidomide patients developed symptom-related bradycardia (21). A likely explanation is that thalidomide inhibited TNF-α expression and activity and led to over activity of the parasympathetic system. Sixty-three (16.3%) patients had HR >100 bpm in our study. Increased HR is a manifestation of impaired cardiac function. The specific mechanism of increased HR in patients with MM is not known but could be a complex matter (17, 18, 23). First, it probably involves both endothelial dysfunction and atrial changes, such as atrial dilatation, sinoatrial node disease, dysfunction of the conduction system, and disruption of normal atrial musculature, which contribute to the development of atrial arrhythmias. In addition, increased adrenergic and increased preload might also lead to atrial arrhythmias induced by electrical remodeling and shortening of the atrial effective refractory period.

Autologous stem cell transplantation (ASCT) is one of the recommended and reasonable choices for MM patients, especially for patients at first relapse and high-risk patients. The incidence of supraventricular arrhythmias during ASCT was approximately 9%. Patients with older age, electrocardiographic abnormalities, and a prior history of arrhythmias had a higher risk of developing supraventricular arrhythmias (24). Atrial fibrillation was one of the most frequent cardiovascular complications in patients with MM receiving ASCT (27%) (24, 25). The percentage of transplantation (9.8%, 38/386) seemed low in our study, although it was affected by many factors, such as physical condition, age, economic inequality, and medical reimbursement policy. Treatment philosophies of physicians were associated with patient choice. Patients in underdeveloped regions had less access to transplantation. All-cause mortality of the patients who received ASCT with HR >100 bpm, 60 ≤ HR ≤ 100 bpm and HR <60 bpm was 66.7% (4/6), 8.0% (2/25) and 0% (0/7), respectively. Similarly, HR showed a close association with all-cause mortality in the three groups. In addition, anemia reflected the higher ISS stages and could lead to arrhythmia. Increased HR was one of the most common symptoms of anemia. The multivariate Cox regression analysis showed that hemoglobin was also an independent risk factor (HR = 0.988, 95% CI: 0.980–0.997). A significant negative correlation between hemoglobin and HR was observed from linear regression analysis (*p* < 0.001). Interestingly, the patients without anemia (hemoglobin >120 g/L) still had an elevated HR in our study. HR was independently associated with all-cause mortality in patients without anemia (*p* < 0.0001, Supplementary Figure 1).

Amyloidosis is a disease in which abnormal immunoglobulin protein deposits aggregates in tissues or organs, such as the kidney, heart, liver, gastrointestinal tract and so on. The incidence of MM-associated light chain amyloidosis is approximately 12–15%, and approximately 38% of newly diagnosed MM patients have clinically occult light chain amyloidosis, which is often overlooked (26). Amyloid deposits can thicken the walls of the heart in myocardial amyloidosis. Restrictive cardiomyopathy is one of the most typical types. Furthermore, the conduction system of the heart is affected, causing arrhythmias and heart block. Endomyocardial biopsy is invasive and difficult to perform. One patient was suspected to have myocardial amyloidosis with typical echocardiography features; however, the patient did not undergo endomyocardial biopsy in the study. In addition, seven patients (1.8%, 7/386) were diagnosed with renal amyloidosis by kidney biopsy in our study.

Previous evidence indicated that sex was one of the important factors of 2-year survival (27). Therefore, the patients were divided into two groups for further analysis of the sex subgroup (10). There was a worse prognosis in the male and female groups with higher HR than in those with a low HR in our study. In addition, the mortality in the female group was higher than that in the male group when the HR was faster than 100 bpm, but no significant effect was observed (*p* > 0.05).

There are several staging systems representing the severity of MM, such as the Durie-Salmon Staging system (DSS), ISS, and Revised International Staging System (R-ISS), at different periods. The DSS classifies patients with MM based on immunoglobulin, hemoglobin, and calcium levels and the number of bone lesions to predict tumor burden and estimate prognosis. ISS, based on serum β-2 microglobulin and albumin, constitutes a potent and powerful MM staging system. Although DSS and ISS are controversial in predicting overall mortality, ISS tends to have a better predictive ability than DSS. The R-ISS staging system, which was published in 2015, affected outcomes in the new medicine era (14). As a simple and convenient tool, ISS is a widely accepted as prognostic staging system for this condition (13, 28) in clinical practice. The R-ISS was based on ISS stages, chromosomal abnormalities, and serum lactate dehydrogenase and is more comprehensive and robust than the ISS. However, new testing technology and the high cost of detection techniques have affected its wide use and acceptance, and not all patients undergo interphase fluorescent *in situ* hybridization (iFISH) detection. This retrospective study was conducted between January 1, 2010, and December 31, 2018. In the study, 76.2% (294/386) of the patients underwent only FISH, 51.6% (199/386) underwent only karyotype analysis, and 49.5% (191/386) underwent both tests. Although there was a significant positive correlation between ISS and HR in linear regression analysis (*p* < 0.001), no difference in all-cause mortality among the three ISS subgroups of patients with HR >100 bpm was observed (*p* > 0.05). In the ISS stage subgroup analysis, patients with fast HR had higher all-cause mortality than those with lower HR in the three ISS groups. Similarly, Markus et al. demonstrated that resting HR was an independent predictor of fatal outcomes in patients with advanced cancer (8).

Advanced age was associated with worse outcomes and probably identified a subpopulation of patients with a higher prevalence of cardiovascular comorbidities. Given the median age at diagnosis of MM, the incorporation of geriatric assessments into treatment decision-making has recently become the focus of investigations (15, 29).

In the subgroup analysis of age, there was a significant correlation of all-cause mortality and HR with age <75 years (*p* < 0.001) but not with age >75 years. This could be because age-related cardiovascular comorbidities attenuate the influence of MM itself on all-cause mortality.

Hypertension is commonly reported in MM patients in clinical trials and may be associated with older age, disease-related complications, and consequences of MM treatments (30–32). A retrospective cohort study reported that hypertension is a risk factor for the development of malignant hypertension in MM patients (33). In our study, however, hypertension was not an independent risk factor for all-cause mortality in MM patients, in part due to the small sample size or various types of BP measurements.

We acknowledge the limitations. First, given the retrospective nature of the study, not all clinical information, such as prescription details of chemotherapy drugs, antibiotics, therapeutic regimen, and strategies and disease state, was available. Second, crossing of survival curves was presented in Figure 4C. It was caused by a variety of reasons, such as the small number of patients (30 cases). In addition, the small study cohort limits our ability to systematically analyze the different antiarrhythmic and antihypertensive therapies prescribed, which might have influenced the results.

## Conclusion

Fast HR was independently associated with high all-cause mortality in MM patients, especially in the group aged <75 years. Our work shows that HR may help to formulate the risk stratification in patients with MM, promoting the management of these high-risk patients with MM and improving their prognosis by reducing cardiac event.

## Data Availability Statement

The raw data supporting the conclusions of this article will be made available by the authors, without undue reservation.

## Ethics Statement

The studies involving human participants were reviewed and approved by the Ethics Committee of the First Affiliated Hospital of Xi'an Jiaotong University. The patients/participants provided their written informed consent to participate in this study.

## Author Contributions

YY and CS contributed to the conception and design of the work and critically revised the manuscript. MT, LC, and JS contributed to acquisition, analysis, or interpretation of data. JW drafted the manuscript. YLo, MW, CS, and YLi critically revised the manuscript. All authors gave final approval and agreed to be accountable for all aspects of the work ensuring integrity and accuracy.

## Conflict of Interest

The authors declare that the research was conducted in the absence of any commercial or financial relationships that could be construed as a potential conflict of interest.

## Publisher's Note

All claims expressed in this article are solely those of the authors and do not necessarily represent those of their affiliated organizations, or those of the publisher, the editors and the reviewers. Any product that may be evaluated in this article, or claim that may be made by its manufacturer, is not guaranteed or endorsed by the publisher.
