# Supplementary material for: Prognostic Potential of Heart Rate and Hypertension in Multiple Myeloma Patients
Source: Front Cardiovasc Med. 2021 Sep 27;8:681484. doi: 10.3389/fcvm.2021.681484 (PMC8502919; doi:10.3389/fcvm.2021.681484)
Supplement: Supplementary file 1 [file Table_1.docx]

**Supplemental Figure legends**

**FIGURE 1.** Kaplan-Meier survival curves according to presence of HR > 100 bpm, 60 ≤ HR ≤ 100 bpm and HR < 60 bpm in patients with hemoglobin > 120 g/L, log-rank *p* < 0.0001. HR, heart rate; bpm, beats per minute.
