# Supplementary figures and images for: Prognostic Potential of Heart Rate and Hypertension in Multiple Myeloma Patients
Source: Front Cardiovasc Med. 2021 Sep 27;8:681484. doi: 10.3389/fcvm.2021.681484 (PMC8502919; doi:10.3389/fcvm.2021.681484)

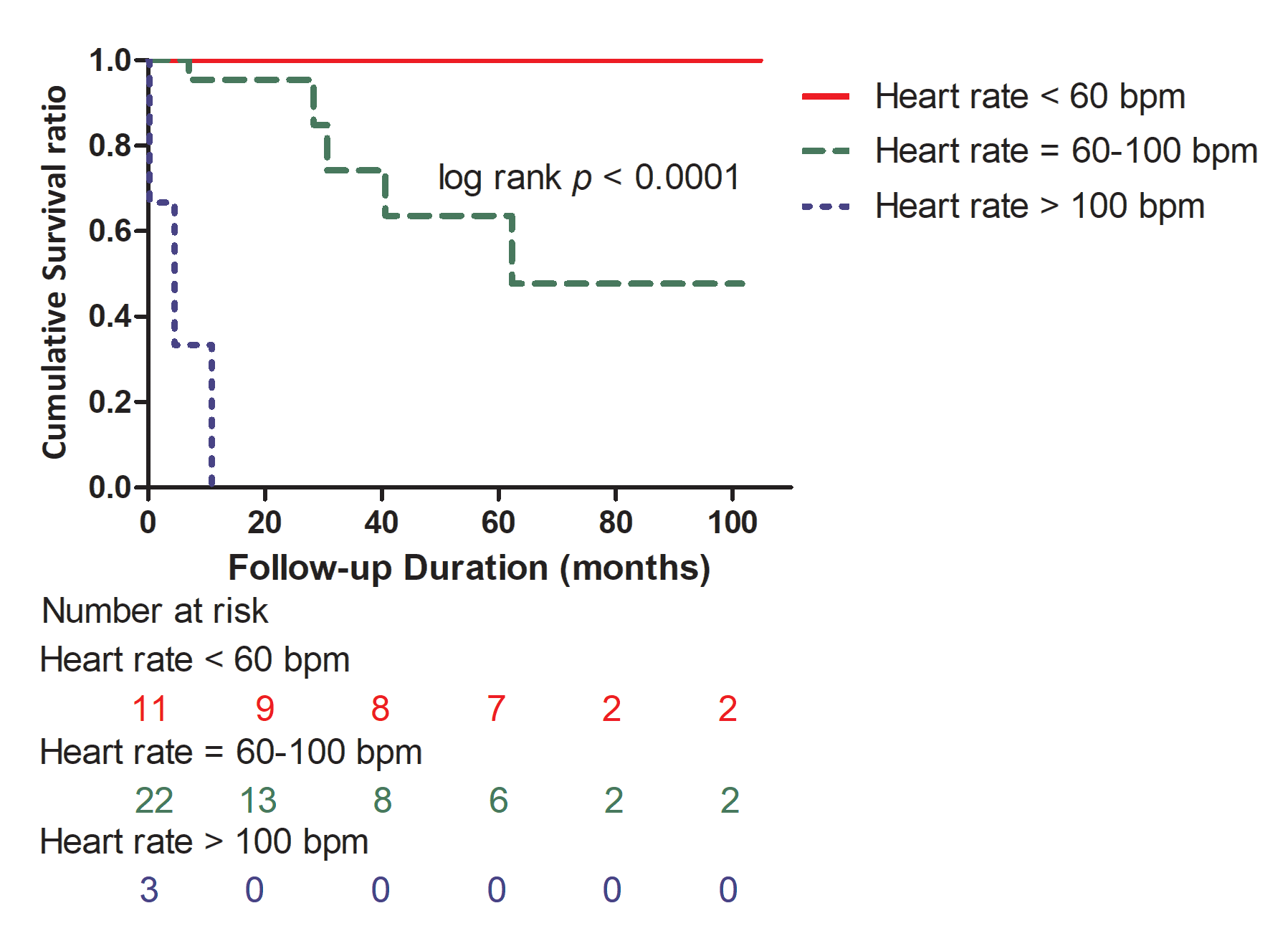

Supplement: Supplementary file 2 [file Image_1.tif]
